# Supplementary figures and images for: A new amino acid substitution in the MvALS1 gene of metsulfuron-methyl resistant biotypes Monochoria vaginalis (Burm. f.) C. Presl from West Java, Indonesia
Source: PLoS One. 2024 Oct 4;19(10):e0308465. doi: 10.1371/journal.pone.0308465 (PMC11451974; doi:10.1371/journal.pone.0308465)

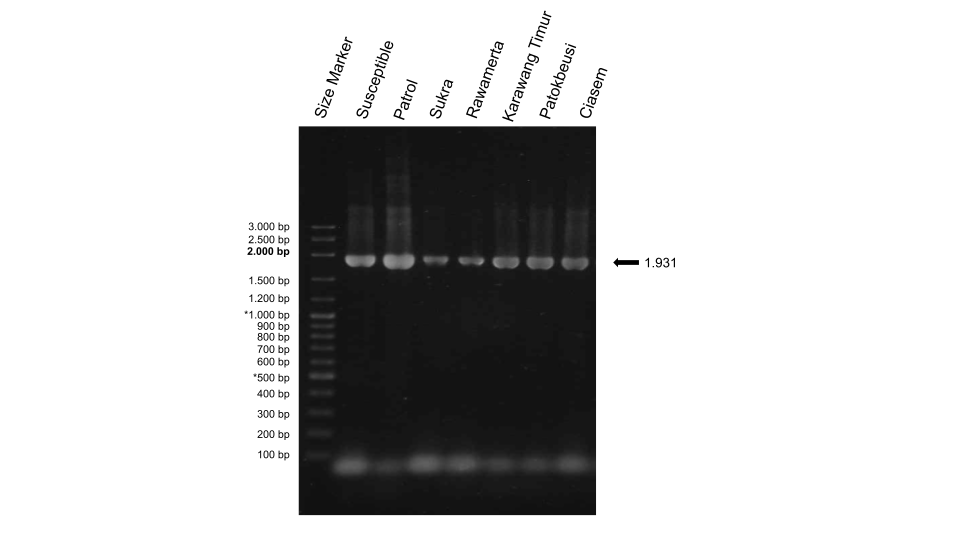

Supplement: S1 Fig — (TIF) [file pone.0308465.s001.tif]

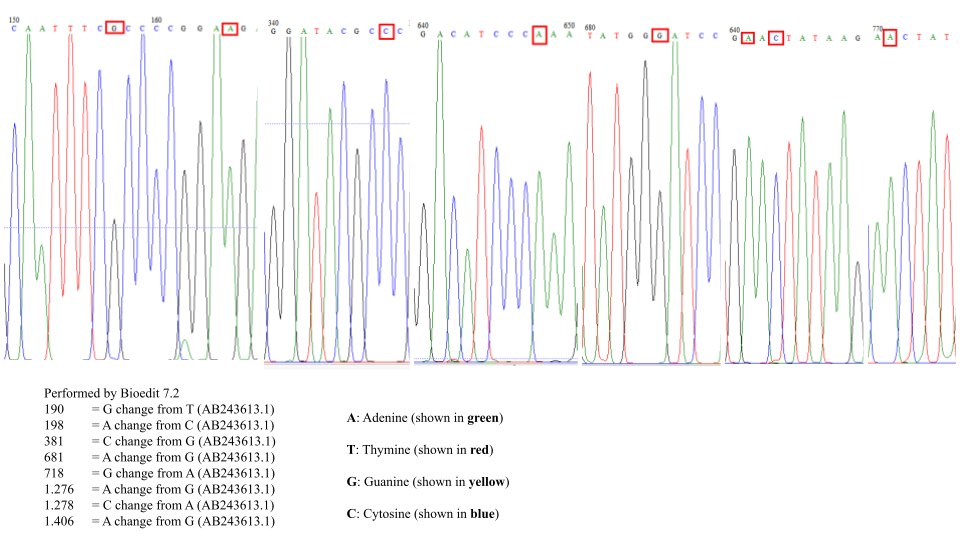

Supplement: S2 Fig — (TIF) [file pone.0308465.s002.tif]
