# Supplementary material for: A new amino acid substitution in the MvALS1 gene of metsulfuron-methyl resistant biotypes Monochoria vaginalis (Burm. f.) C. Presl from West Java, Indonesia
Source: PLoS One. 2024 Oct 4;19(10):e0308465. doi: 10.1371/journal.pone.0308465 (PMC11451974; doi:10.1371/journal.pone.0308465)
Supplement: S1 Table — (PDF) [file pone.0308465.s003.pdf]

S1 Table. Purification of *M. vaginalis* results of a DNA spectrophotometer.

Biotype	Absorbance				Concentration	DNA Quality	
	A_{230}	A_{260}	A_{280}	A_{320}	(µg mL^{-1})	$A_{260/280}$	$A_{260/230}$
**Susceptible**	0.164	0.043	0.033	0.021	22.240	1.863	0.153
**Patrol**	0.173	0.085	0.068	0.049	37.450	1.902	0.296
**Sukra**	0.202	0.093	0.080	0.058	35.200	1.643	0.239
**Rawamerta**	0.192	0.038	0.035	0.032	6.122	2.308	0.0376
**Karawang Timur**	0.179	0.084	0.074	0.052	32.450	1.426	0.250
**Patokbeusi**	0.266	0.089	0.078	0.060	29.490	1.624	0.141
**Ciasem**	0.2078	0.0699	0.0601	0.0444	26.020	1.624	0.156

Concentration (µg mL^{-1}) Formula: $(A_{260}-A_{320})*(50/0.049)$ a µDrop plate: 0.049 cm pathlength
Purity (A_{260}/A_{280}) Formula: $(A_{260}-A_{320})/(A_{280}-A_{320})$ Source step: Blank Subtraction
Purity (A_{260}/A_{230}) Formula: $(A_{260}-A_{320})/(A_{230}-A_{320})$ Source step: Blank Subtraction
