# Supplementary material for: A new amino acid substitution in the MvALS1 gene of metsulfuron-methyl resistant biotypes Monochoria vaginalis (Burm. f.) C. Presl from West Java, Indonesia
Source: PLoS One. 2024 Oct 4;19(10):e0308465. doi: 10.1371/journal.pone.0308465 (PMC11451974; doi:10.1371/journal.pone.0308465)
Supplement: S2 Table — (PDF) [file pone.0308465.s004.pdf]

S2 Table. Origin Pro Data and Analysis on Metsulfuron-Methyl GR_{50}.

Dosage (g a.i. ha^{-1})	Biotype						
	Susceptible (%)	Patrol (%)	Sukra (%)	Rawamerta (%)	Karawang Timur (%)	Patokbeusi (%)	Ciasem (%)
**0**	0.00d	0.00e	0.00f	0.00f	0.00d	0.00f	0.00d
**1**	74.58c	52.29d	35.71e	26.67e	60.00c	37.86e	63.60c
**2**	87.29b	73.75c	57.14d	38.33d	80.00b	44.76d	83.52b
**4**	93.65a	82.81b	76.79c	48.33c	91.67a	49.88c	92.72a
**8**	93.65a	86.88b	85.71b	63.33b	91.67a	82.74b	92.72a
**16**	93.65a	93.44a	91.07a	81.67a	91.67a	89.64a	92.72a
**32**	93.65a	93.44a	92.86a	90.00a	93.33a	93.10a	92.72a

Note: significant differences within a single column are indicated by different letters, as determined by the Duncan multiple range test at a significance level of α=0.05. The percentage of damage (%) is calculated using the formula (1 - (P/K)) × 100%, where P represents the dry weight of weeds treated with herbicide, and K represents the dry weight of control weeds.

Model	DoseResp
Equation	$y = A_1 + (A_2-A_1)/(1 + 10^{((LOGx_0-x)*p)})$

Biotype	A_1 Value	A_1 Standard Error	A_2 Value	A_2 Standard Error	$LOGx_0$ Value	$LOGx_0$ Standard Error	p Value	p Standard Error	span Value	GR_{50} Value
**Susceptible**	0	0	93.65	0	-0.28839	0.16926	2.03936	1.01199	93.65	0.51477
**Patrol**	0	0	93.65	0	-0.08746	0.06426	1.33248	0.2494	93.65	0.8176
**Sukra**	0	0	93.65	0	0.15344	0.03762	1.41785	0.1866	93.65	1.42377
**Rawamerta**	0	0	93.65	0	0.48941	0.04111	0.9752	0.09995	93.65	3.08609
**Karawang Timur**	0	0	93.65	0	-0.1344	0.06568	1.84199	0.4657	93.65	0.73384
**Patokbeusi**	0	0	93.65	0	0.3019	0.04262	1.04642	0.11789	93.65	2.004
**Ciasem**	0	0	93.65	0	-0.15497	0.07261	2.08344	0.62985	93.65	0.6999
