# Supplementary material for: A new amino acid substitution in the MvALS1 gene of metsulfuron-methyl resistant biotypes Monochoria vaginalis (Burm. f.) C. Presl from West Java, Indonesia
Source: PLoS One. 2024 Oct 4;19(10):e0308465. doi: 10.1371/journal.pone.0308465 (PMC11451974; doi:10.1371/journal.pone.0308465)
Supplement: S3 Table — (PDF) [file pone.0308465.s005.pdf]

S3 Table. Estimated Enzyme Commission (EC) numbers and active site residues.

Rank	CscoreEC	PDB Hit	TM-score	RMSDa	IDENa	Cov.	EC Number	Predicted Active Site Residues
1	680	1ybhA	902	0.75	746	906	2.2.1.6	615, 457, 483, 485
2	536	2ji6A	773	2.65	226	822	4.1.1.8	458, 483, 485
3	520	2panA	841	1.95	301	872	4.1.1.47	483
4	381	2q5IA	723	3.88	205	819	4.1.1.74<br>4.1.1.43	484, 485
5	375	3flmA	689	3.80	156	787	2.2.1.9	481

[a]CscoreEC: Confidence score (from 0 to 1) for predicting the Enzyme Commission (EC) number. More scores signify more trustworthy forecasts.
[b]TM-score: Quantification of query and template proteins' global structural similarity.
[c]RMSDa: RMSD between residues that TM-align structurally aligned.
[d]IDENa: Percentage sequence identity in the structurally aligned region.
[e]Cov: Coverage of global structural alignment, calculated by dividing the total number of residues with structural alignment by the query protein's length.
