# Supplementary material for: A new amino acid substitution in the MvALS1 gene of metsulfuron-methyl resistant biotypes Monochoria vaginalis (Burm. f.) C. Presl from West Java, Indonesia
Source: PLoS One. 2024 Oct 4;19(10):e0308465. doi: 10.1371/journal.pone.0308465 (PMC11451974; doi:10.1371/journal.pone.0308465)
Supplement: Attachment — Submitted filename: PONE-D-24-02160_reviewer.pdf [file pone.0308465.s006.pdf]

# PLOS ONE

# A New Amino Acid Substitution in the MvALS1 Gene of Metsulfuron-Methyl Resistant Biotypes Monochoria vaginalis (Burm. f.) C. Presl From West Java, Indonesia

--Manuscript Draft--

| | |
|---|---|
| **Manuscript Number:** | PONE-D-24-02160 |
| **Article Type:** | Research Article |
| **Full Title:** | A New Amino Acid Substitution in the MvALS1 Gene of Metsulfuron-Methyl Resistant Biotypes Monochoria vaginalis (Burm. f.) C. Presl From West Java, Indonesia |
| **Short Title:** | A Novel Amino Acid Substitution in the MvALS1 Gene of Metsulfuron-Methyl Resistant Biotypes M. vaginalis |
| **Corresponding Author:** | Kansa Dianti Putri, S.Agr<br>IPB University: Institut Pertanian Bogor<br>Karawang, Indonesia INDONESIA |
| **Keywords:** | bio-assay; Monochoria vaginalis; metsulfuron-methyl; target site resistance |
| **Abstract:** | The most bothersome weed in rice fields in the Indonesian province of West Java is Monochoria Vaginalis (Burm. F.) C. Presl, an aquatic herbaceous plant. Metsulfuron-methyl has long been used in wetland rice in West Java with a high enough intensity. However, the case of Monochoria Vaginalis resistance to metsulfuron-methyl herbicides in Indonesia has not been widely reported and investigated. The study aims to (1) classify the resistance level of M. vaginalis toward metsulfuron-methyl, (2) identify Target Site Resistance (TSR) mechanism mutations in the MvALS1 gene of the resistant biotype of M. vaginalis. The Whole Plant Pot Test method was utilized to assess the resistance level of Monochoria vaginalis. Following that, all samples were subjected to DNA sequencing using the PCR method to identify mutations in the MvALS1 gene from the resistant biotype. The result showed that Monochoria vaginalis from Rawamerta, Karawang showed a moderate level of resistance to metsulfuron-methyl with a resistance ratio of 6.00, Patokbeusi, Subang showed a low level of resistance to metsulfuron-methyl with a resistance ratio of 3.89, compared to susceptible Monochoria vaginalis. Nucleotide base alignment in the MvALS1 gene revealed that base substitutions occurred in the Monochoria vaginalis biotype from Rawamerta, resulting in 5 amino acid substitutions: Ser-64-Ala, Asp-66-Glu, Asn-240-Asp, Glu-426-Asn, and Ser-469-Asn, Patokbeusi: Ser-64-Ala, Asp-66-Glu, Asn-240-Asp, and Ser-469-Asn and Sukra: Ser-64-Ala, Asp-66-Glu, and Asn-240-Asp. This study confirms for the first time that Ser-64-Ala, Asn-240-Asp, and Glu-426-Asn amino acid mutations were found in cases of M. vaginalis resistance to metsulfuron-methyl (ALS inhibitor). |
| **Order of Authors:** | Kansa Dianti Putri, S.Agr |
| | Dwi Guntoro |
| | Sintho Wahyuning Ardie |
| | Hariyadi Hariyadi |
| **Additional Information:** | |
| **Question** | **Response** |
| **Financial Disclosure**<br><br>Enter a financial disclosure statement that describes the sources of funding for the work included in this submission. Review the submission guidelines for detailed requirements. View published research articles from *PLOS ONE* for specific examples. | Yes |

| | |
|---|---|
| This statement is required for submission and **will appear in the published article** if the submission is accepted. Please make sure it is accurate.<br><br>**Funded studies**<br>Enter a statement with the following details:<br>• Initials of the authors who received each award<br>• Grant numbers awarded to each author<br>• The full name of each funder<br>• URL of each funder website<br>• Did the sponsors or funders play any role in the study design, data collection and analysis, decision to publish, or preparation of the manuscript?<br><br>Did you receive funding for this work? | |
| Please add funding details.<br>as follow-up to "**Financial Disclosure**<br><br>Enter a financial disclosure statement that describes the sources of funding for the work included in this submission. Review the submission guidelines for detailed requirements. View published research articles from *PLOS ONE* for specific examples.<br><br>This statement is required for submission and **will appear in the published article** if the submission is accepted. Please make sure it is accurate.<br><br>**Funded studies**<br>Enter a statement with the following details:<br>• Initials of the authors who received each award<br>• Grant numbers awarded to each author<br>• The full name of each funder<br>• URL of each funder website<br>• Did the sponsors or funders play any role in the study design, data collection and analysis, decision to publish, or preparation of the manuscript? | Kansa Dianti Putri 202207110310741 was granted funding by LPDP - Educational Fund Management Institution to support the published article (https://lpdp.kemenkeu.go.id/). The funding was given to the scholarship recipient who was accepted into the Q1 Journal, with no involvement in the paper itself. |

| | |
|---|---|
| Did you receive funding for this work?" | |
| Please select the country of your main research funder (please select carefully as in some cases this is used in fee calculation).<br>as follow-up to "**Financial Disclosure**<br><br>Enter a financial disclosure statement that describes the sources of funding for the work included in this submission. Review the submission guidelines for detailed requirements. View published research articles from *PLOS ONE* for specific examples.<br><br>This statement is required for submission and **will appear in the published article** if the submission is accepted. Please make sure it is accurate.<br><br>**Funded studies**<br>Enter a statement with the following details:<br>• Initials of the authors who received each award<br>• Grant numbers awarded to each author<br>• The full name of each funder<br>• URL of each funder website<br>• Did the sponsors or funders play any role in the study design, data collection and analysis, decision to publish, or preparation of the manuscript?<br><br>Did you receive funding for this work?" | INDONESIA - ID |
| **Competing Interests**<br><br>Use the instructions below to enter a competing interest statement for this submission. On behalf of all authors, disclose any competing interests that could be perceived to bias this work—acknowledging all financial support and any other relevant financial or non-financial competing interests.<br><br>This statement is required for submission | The authors have declared that no competing interests exist. |

| | |
|---|---|
| and **will appear in the published article** if the submission is accepted. Please make sure it is accurate and that any funding sources listed in your Funding Information later in the submission form are also declared in your Financial Disclosure statement.<br><br>View published research articles from *PLOS ONE* for specific examples.<br><br>**NO authors have competing interests**<br><br>Enter: *The authors have declared that no competing interests exist.*<br><br>**Authors with competing interests**<br><br>Enter competing interest details beginning with this statement:<br><br>*I have read the journal's policy and the authors of this manuscript have the following competing interests: [insert competing interests here]*<br><br>* typeset | |
| **Ethics Statement**<br><br>Enter an ethics statement for this submission. This statement is required if the study involved:<br><br>• Human participants<br>• Human specimens or tissue<br>• Vertebrate animals or cephalopods<br>• Vertebrate embryos or tissues<br>• Field research<br><br>Write "N/A" if the submission does not require an ethics statement.<br><br>General guidance is provided below. Consult the submission guidelines for detailed instructions. **Make sure that all information entered here is included in the Methods section of the manuscript.** | N/A |

| | |
|---|---|
| **Format for specific study types**<br><br>**Human Subject Research (involving human participants and/or tissue)**<br>• Give the name of the institutional review board or ethics committee that approved the study<br>• Include the approval number and/or a statement indicating approval of this research<br>• Indicate the form of consent obtained (written/oral) or the reason that consent was not obtained (e.g. the data were analyzed anonymously)<br><br>**Animal Research (involving vertebrate animals, embryos or tissues)**<br>• Provide the name of the Institutional Animal Care and Use Committee (IACUC) or other relevant ethics board that reviewed the study protocol, and indicate whether they approved this research or granted a formal waiver of ethical approval<br>• Include an approval number if one was obtained<br>• If the study involved *non-human primates*, add *additional details* about animal welfare and steps taken to ameliorate suffering<br>• If anesthesia, euthanasia, or any kind of animal sacrifice is part of the study, include briefly which substances and/or methods were applied<br><br>**Field Research**<br><br>Include the following details if this study involves the collection of plant, animal, or other materials from a natural setting:<br>• Field permit number<br>• Name of the institution or relevant body that granted permission | |
| **Data Availability**<br><br>Authors are required to make all data underlying the findings described fully available, without restriction, and from the time of publication. PLOS allows rare exceptions to address legal and ethical concerns. See the PLOS Data Policy and FAQ for detailed information. | Yes - all data are fully available without restriction |

| | |
|---|---|
| A Data Availability Statement describing where the data can be found is required at submission. Your answers to this question constitute the Data Availability Statement and **will be published in the article**, if accepted.<br><br>**Important:** Stating 'data available on request from the author' is not sufficient. If your data are only available upon request, select 'No' for the first question and explain your exceptional situation in the text box.<br><br>Do the authors confirm that all data underlying the findings described in their manuscript are fully available without restriction? | |
| **Describe where the data may be found in full sentences. If you are copying our sample text, replace any instances of XXX with the appropriate details.**<br><br>• If the data are **held or will be held in a public repository**, include URLs, accession numbers or DOIs. If this information will only be available after acceptance, indicate this by ticking the box below. For example: *All XXX files are available from the XXX database (accession number(s) XXX, XXX.).*<br>• If the data are all contained **within the manuscript and/or Supporting Information files**, enter the following: *All relevant data are within the manuscript and its Supporting Information files.*<br>• If neither of these applies but you are able to provide **details of access elsewhere**, with or without limitations, please do so. For example:<br><br>*Data cannot be shared publicly because of [XXX]. Data are available from the XXX Institutional Data Access / Ethics Committee (contact via XXX) for researchers who meet the criteria for access to confidential data.*<br><br>*The data underlying the results presented in the study are available from (include the name of the third party* | All relevant data are within the manuscript and its Supporting Information files. |

| | |
|---|---|
| *and contact information or URL).*<br>• This text is appropriate if the data are owned by a third party and authors do not have permission to share the data.<br><br>* typeset | |
| Additional data availability information: | |

Manuscript
Click here to access/download;Manuscript;plos_latex_template 16012024.docx

# A New Amino Acid Substitution in the *MvALS1* Gene of Metsulfuron-Methyl Resistant Biotypes *Monochoria vaginalis* (Burm. f.) C. Presl From West Java, Indonesia

Kansa Dianti Putri[1*], Dwi Guntoro[1], Sintho Wahyuning Ardie[1], Hariyadi[1]

**1** Department of Agronomy and Horticulture, IPB University, Bogor, Indonesia

* kansadianti@apps.ipb.ac.id

## Abstract

The most bothersome weed in rice fields in the Indonesian province of West Java is *Monochoria Vaginalis* (Burm. F.) C. Presl, an aquatic herbaceous plant. Metsulfuron-methyl has long been used in wetland rice in West Java with a high enough intensity. However, the case of *Monochoria Vaginalis* resistance to metsulfuron-methyl herbicides in Indonesia has not been widely reported and investigated. The study aims to (1) classify the resistance level of *M. vaginalis* toward metsulfuron-methyl, (2) identify Target Site Resistance (TSR) mechanism mutations in the *MvALS1* gene of the resistant biotype of *M. vaginalis*. The Whole Plant Pot Test method was utilized to assess the resistance level of *Monochoria vaginalis*. Following that, all samples were subjected to DNA sequencing using the PCR method to identify mutations in the *MvALS1* gene from the resistant biotype. The result showed that *Monochoria vaginalis* from Rawamerta, Karawang showed a moderate level of resistance to metsulfuron-methyl with a resistance ratio of 6.00, Patokbeusi, Subang showed a low level of resistance to metsulfuron-methyl with a resistance ratio of 3.89, compared to susceptible *Monochoria vaginalis*. Nucleotide base alignment in the *MvALS1* gene revealed that base substitutions occurred in the *Monochoria vaginalis* biotype from Rawamerta, resulting in 5 amino acid substitutions: Ser-64-Ala, Asp-66-Glu, Asn-240-Asp, Glu-426-Asn, and Ser-469-Asn, Patokbeusi: Ser-64-Ala, Asp-66-Glu, Asn-240-Asp, and Ser-469-Asn and Sukra: Ser-64-Ala, Asp-66-Glu, and Asn-240-Asp. This study confirms for the first time that Ser-64-Ala, Asn-240-Asp, and Glu-426-Asn amino acid mutations were found in cases of *M. vaginalis* resistance to metsulfuron-methyl (ALS inhibitor).

## Introduction

Consumption of West Java domestic rice is the highest in Indonesia 3.85 million tons or about 18.64% of total household consumption [1]. The three districts with the largest rice production in West Java namely Indramayu Regency 1.42 million tons, Karawang Regency 1.10 million tons and Subang Regency 1.02 million tons [2]. However, some efforts to maintain and increase rice yields often encounter several problems, including competition between rice plants and weeds.

Weed species are the most dominant in rice fields are *Monochoria vaginalis*, *Echinochloa glabrescens*, and *Ludwigia octovalvis* [3]. *Monochoria vaginalis* is possible causing rice yield to decrease by up to 82% and *M. vaginalis* in density 150 $m^{-2}$ can reduce rice yield by about 25% in Indonesia [4, 5]. The highest dominance of weeds in rice fields in Subang, West Java is *Monochoria vaginalis* with a total value dominance (NJD) is 35.89% [6] (Evar et al. 2022). *Monochoria vaginalis* spreads by vegetative propagation and is a C3 plant, similar to rice [7].

Currently, herbicides are widely used to control weeds in Indonesia. Farmers use herbicides due to a lack of workers willing to work in agricultural land and the difficulty in finding personnel to control weeds mechanically. The chemical use of herbicides is one way

to control weeds in rice cultivation. The utilization of herbicides is 80% more profitable than conventional weeding [8]. Metsulfuron-methyl (sulfonylurea group) is a broadleaf weed control herbicide that inhibits enzim acetolactate synthase (ALS). It is widely used in rice fields in Indonesia. ALS (E.C. 2.2.1.6) catalyzes the first step in the biosynthesis of the branched-chain amino acids valine (Val), leucine (Leu), and isoleucine (Ile). [9]. However, the extensive use of herbicides will cause weed resistance. Weed resistance occurs frequently over a long period due to the utilization of the same herbicide or herbicide with the same mode of action [10]. *M. vaginalis* from Karawang and Subang (Indonesia) are known to exhibit metsulfuron-methyl resistance with R/S of 3.05 and 2.14, meaning the dose used to inhibit 50% of the population exceeds the recommended dose of 20 g $ha^{-1}$ [6].

The resistance mechanism is based on the level of involvement of the target protein, specifically target site resistance (TSR), which is related to mutations at the target sites of herbicide action and resistance to non-target related (NTSR) location with secondary metabolic pathways [11]. The plant ALS enzyme, encoded by the Als gene, includes at least three copies of *MkALS* in *Monochoria korsakowii* and five copies of *MvALS* in *M. vaginalis* [12]. Point mutations in the gene encoding ALS are thought to cause herbicide resistance. A Japanese population of M. *vaginalis* was found to be resistant to the herbicide, and the changes in the amino acids Pro-197-Ser and Asp-376-Glu were caused by two point mutations in the *MvALS1* gene. *MvALS1* gene expression was reported to be higher than other *MvALS* in *M. vaginalis* [13], thus mutations in *MvALS1* gene are likely to cause herbicide resistance through the TSR mechanism. Weed resistance to herbicides may cause problems later due to lack of knowledge and database, such as increasing herbicide dosage and intensity, which would encourage Weed herbs to thrive and become more resilient species. Therefore, this experiment was conducted to (1) classify the resistance level of *M. vaginalis* toward metsulfuron-methyl, (2) identify Target Site Resistance (TSR) mechanism mutations in the *MvALS1* gene of the resistant biotype of *M. vaginalis*.

# Materials and methods

## Plant materials and growth conditions

Seeds of suspected resistant *M. vaginalis*. populations were collected from rice fields in Patrol (6°18'53.0"S 108°00'19.2"E), Sukra (6°18'41.2"S 107°58'46.2"E), Rawamerta (6°15'12.7"S 107°20'30.3"E), Karawang Timur (6°18'22.0"S 107°19'35.4"E), Patokbeusi (6°20'54.6"S 107°38'51.4"E), and Ciasem (6°18'30.6"S 107°42'56.5"E) districts. *M. vaginalis*. seeds were also obtained from herbicide-untreated paddy fields in Tambakdahan (6°21'39.9"S 107°48'07.8"E), subsequently referred to as susceptible biotype. The location was chosen based on reports from farmers in the three largest rice producing districts in West Java [2], with a location map in S1 Fig. The Whole Plant Pot Test system [14] (Burgos, 2015) is the method used in this study. The plants were cultivated in plastic pots (20 cm in diameter) that were filled with rice paddy field soil. The experimental design consisted of a split-plot design with two factors and four replications for each experiment. The first factor was the origin of weeds, as the main plot contained seven weed locations, and the second factor was the metsulfuron-methyl 20% dose.

## Dose-response experiment

Seedlings have been thinned to 20 plants pot-1 before herbicide treatment. At the 2- to 3-leaf stage, herbicides were applied using a Bengawan Solo 425 Compression Sprayer Manual 15 liter, Lurmark flat fan green nozzle 1.2 litre $minute^{-1}$. Metsulfuron-methyl was applied at 0, 1, 2, 4, 8, 16, and 32 g a.i. $ha^{-1}$. These rates are equivalent to 0, 0.25 0.5, 1, 2, 4, and 8 times of the recommended field rate of the products. The recommended rate of metsulfuron-methyl is 4 g a.i. $ha^{-1}$, respectively. The plants were cut on the soil surface twenty-one days after treatment (DAT), oven-dried for 48 h at 80°C [15], and shoot dry weights were recorded.

### Data analysis

The whole-plant dose-response data were exposed to ANOVA utilizing SPSS v. 25.0. Non-linear regressions of equation (1) were used to analyze data [16]:

$$y = C + \frac{D - C}{1 + (x/I_{50})^b} \quad (1)$$

where: C= lower limit, D= upper limit, b= slope, and $I_{50}$= a dosage that gives a 50% response

An analysis of the dose-response curves was performed using Origin Pro 2016 [17]. For each herbicide, the Resistance-Susceptible (R/S) ratio was determined as follows: $GR_{50}$ of the resistant (R) population/$GR_{50}$ of the susceptible (S) population. According to [18], herbicide sensitivity is further divided into five classes: sensitive (R/S< 2.0), reduced sensitivity (R/S= 2.0–2.9), low resistance (R/S= 3.0–4.9), high resistance (R/S= 10.0-68.1), and high resistance (R/S> 68.2).

### Isolation of DNA and gene sequence

Genomic DNA was isolated from the leaves of susceptible and resistant biotypes of *M. vaginalis*. Leaves weighing 0.1-0.5 g were mashed together with 700 L of DNA extraction buffer solution [100 mM Tris HCl (pH 8), 1.4 M NaCl, 20 mM EDTA, 2% CTAB, and 1% PVP] [19]. The DNA quality was determined using 1% agarose, and the quantity of DNA was tested using a Thermo ScientificTM DropTM and Multiskan SkyHigh Microplate Spectrophotometer, with the results shown in S1 Table. A primer set 5'-ATGGCTGCTTCGAAGCCCTCTCCATT-3' (forward) and 5' ACTAGTGCACTGTGCTCCCATCTCCAT-3' (reverse) was used to amplify DNA with a length of 1.930 bp at the position between 1 base and 1.931 base in the gene encoding ALS1 (GenBank accession number AB243613.1). Amplification was carried out by PCR in a total volume of 50 µL containing 25 µL 2x KAPA2G Fast ReadyMix Kit, 2.5 µL forward and reverse primers with a concentration of 10 µM each, 12.5 µL of M.vaginalis genomic DNA with a concentration of 2 ng $\mu L^{-1}$ and 7.5 µL Nuclease Free Water (NFW). The PCR conditions were as follows: denaturation at 94 °C for 2 min; 40 cycles of 94 °C for 5 s, 60 °C for 30 s; elongation temperature at 72 °C for 1 min, and final extension at 72 °C for 10 min. The amplification results were analyzed by electrophoresis of 5 µL of PCR product on a 1.5% (w/v) agarose gel at 90 V for 85 min in 1x TAE buffer (Tris-acetate-EDTA). The gel resulting from electrophoresis was then stained with ethidium bromide (0.5 µg/mL) for 10 seconds and continued with visualization using a UV transilluminator (Gel Doc EZTM, Bio-Rad, United States). Amplification of the *MvALS1* gene produced an amplicon of approximately 1.930 bp in each of the seven *M.vaginalis* biotypes in S2 Fig. Nucleotide base tracing was carried out using the Sanger method by the service company PT Genetica Science Indonesia. Alignment of nucleotide base sequences was carried out using the MUSCLE algorithm in Bioedit 7.2.

## Results

### Sensitivity to ALS herbicides

The dosage response curve (Fig 1) was used to obtain the $GR_{50}$ value for each treatment of population origin and herbicide level. The sensitive biotype's $GR_{50}$ value was 0.51 g a.i. $ha^{-1}$. Meanwhile, two of the six resistant *Monochoria vaginalis* populations, Rawamerta and Patokbeusi, demonstrated resistance to the herbicide metsulfuron-methyl, with a $GR_{50}$ value of 3.09 g a.i. $ha^{-1}$ (dosage 0.77 times higher than the recommended dose) for Rawamerta and $GR_{50}$ 2.00 g a.i. $ha^{-1}$ (dosage 0.50 times higher than recommended dose) for Patokbeusi. The

survival percentage for Rawamerta and Patokbeusi biotypes was thought to be higher than the required dose of metsulfuron-methyl, which is >4 g a.i. $ha^{-1}$ to kill 50% of the *Monochoria vaginalis* population.

**Fig 1. Inhibition curves by metsulfuron-methyl from seven populations of *M. vaginalis*.** Lines are the reaction bends foreseen by non-linear regression; symbols represent shares of average survival, upheld the untreated controls.

Based on the results of previous research in Indonesia, the $GR_{50}$ value of *E. crus-galli* from East Karawang was 7.59 g a.i. $ha^{-1}$ metsulfuron-methyl. The $GR_{50}$ value of metsulfuron-methyl was 1.90 times higher (7.59 g a.i. $ha^{-1}$) than the recommended application dose (4 g a.i. $ha^{-1}$) [20], and the resistance factor against *Chamaesyce maculate* from Georgia was 90 times higher than sensitive biotypes [21]. Then, in Subang, *M. vaginalis* exhibited a $GR_{50}$ value of 51.93 g a.i. $ha^{-1}$ bensulfuron-methyl (sulfenylurea), which was 13 times higher than the recommended dose (4 g a.i. $ha^{-1}$) [15]. In addition, *M. vaginalis* had a $GR_{50}$ value of 9.50 g a.i. $ha^{-1}$ bensulfuron-methyl (sulfenylurea) in Korea, which was 2.38 times higher than the recommended dose (4 g a.i. $ha^{-1}$) [22].

## Confirmation of resistance

The comparison value between $GR_{50}$ *Monochoria vaginalis* exposed to herbicides and $GR_{50}$ *Monochoria vaginalis* not exposed to herbicides (sensitive) was used to calculate a resistance ratio, and the resistance ratio value is used to assess weed resistance to herbicides [18]. Table 1 shows the resistance level of *Monochoria vaginalis* accessions to the herbicide metsulfuron-methyl. Patrol, East Karawang, and Ciasem biotypes were sensitive to metsulfuron-methyl, with R/S values of 1.59, 1.43, and 1.36, respectively. Meanwhile, *Monochoria vaginalis* from Rawamerta exhibited moderate resistance to metsulfuron methyl (R/S 6.00), indicating that it was 6.00 times more resistant than sensitive *Monochoria vaginalis*. *Monochoria vaginalis* from Patokbeusi showed low resistance to metsulfuron methyl (R/S 3.89), but *Monochoria vaginalis* from Sukra demonstrated low sensitivity (R/S 2.77). This low resistance occurred because it was discovered that raising the dose may still produce a decrease in the average dry weight of *Monochoria vaginalis* from East Karawang. Thus far, 14.29% of the *Monochoria vaginalis* population from West Java were moderate resistance (R/S=5.0-9.9), 14.29%, low resistance (R/S=3.0-4.9), 14.29% decreased sensitivity (R/S= 2-2.9) and 57.14% sensitive (R/S=<2) to metsulfuron-methyl.

**Table 1. Metsulfuron-methyl dose needed to kill 50% of plants ($GR_{50}$) and R/S ratios obtained from the curves of population S and R *M. vaginalis*.**

| Biotype | $GR_{50}$ (g b.a $ha^{-1}$) | *Adj. R-square* | R/S | Resistance Category [18] |
|---|---|---|---|---|
| **Sensitive** | 0.51 | 0.98 | - | - |
| **Patrol** | 0.82 | 0.98 | 1.59 | Susceptible |
| **Sukra** | 1.42 | 0.99 | 2.77 | Reduced Sensitivity |
| **Rawamerta** | 3.09 | 0.95 | 6.00 | Moderate Resistance |
| **Karawang Timur** | 0.73 | 0.98 | 1.43 | Susceptible |
| **Patokbeusi** | 2.00 | 0.86 | 3.89 | Low Resistance |
| **Ciasem** | 0.70 | 0.99 | 1.36 | Susceptible |

[a]$GR_{50}$ refers to the measurements of herbicide success in retaining 50% dry weight and is expressed as active ingredient grams per hectare (g a.i. $ha^{-1}$). The results of the nonlinear regression analysis of the log-logistic model in S2 Table.
[b]R/S, index Resistance was computed as the ratio of $GR_{50}$ R to $GR_{50}$ S.

The visual representation of the differences in metsulfuron-methyl resistance levels from each location is shown in (Fig 2). Visually, the genotype from Rawamerta, Karawang with R/S 6.00 when the recommended dose (4 g a.i. $ha^{-1}$) was applied had higher growth than other genotypes. Other researchers have discovered *M. vaginalis* populations resistant to

bensulfuron-methyl (sulfonylurea). Indonesia had an R/S ratio of 31.28, while South Korea had an R/S ratio of 9.50 [15, 22]. Furthermore, *E. crus-galli* from East Karawang, Indonesia developed resistance to metsulfuron-methyl (sulfenylurea) for the first time in the world, with an R/S of 5.88 [20].

**Fig. 2. The growth of resistance (R) and susceptible (S) *M. vaginalis* biotypes**

The *Monochoria vaginalis* weed population from Rawamerta is classified as moderately resistant with a resistance ratio value of 6.00, Patokbeusi as low resistance with a resistance ratio value of 3.89, and Sukra as decreasing sensitivity with a resistance ratio value of 2.77 to the herbicide metsulfuron-methyl (Table 4.3). Metsulfuron-methyl herbicide, works by transporting it from the xylem and phloem to all weed tissues and acting as an inhibitor of the aceto-lactate synthase enzyme, which blocks the production of the three amino acids valine, leucine, and isoleucine, thereby inhibiting plant shoot and root cell division [23]. ALS reduces the active molecule thiamine diphosphate (ThDP) in cells and inhibits the effect of herbicide interventions. ThDP is a cofactor for the ALS enzyme, which produces energy for cell division and all metabolic processes in plant cells. Reducing ThDP prevents the amino acid chains leucine, isoleucine, and valine from forming. If ThDP requirements are not met, growth can be slowed by symptoms such as yellowing, necrosis, and inhibited root development [24].

## Identification of the mutation in *MvALS1* genes

The nucleotide sequences of the 1.930 bp region from the resistant *M. vaginalis* biotype differed by a single nucleotide substitution from the susceptible biotype. The alignment of the *MvALS1* gene sequence in the seven *M. vaginalis* genotypes reveals changes in nucleotide bases for the third genotype (Fig 3). First, *M. vaginalis* from Rawamerta was classified as moderately resistant to metsulfuron-methyl due to changes in nucleotide bases at 8 positions: 190, 198, 381, 681, 718, 1,276, 1,278, and 1,406. Second, *M. vaginalis* from Patokbeusi had low resistance and experienced changes in nucleotide bases at six sites: 190, 198, 718, 1,276, 1,278, and 1406. Finally, *M. vaginalis* from Sukra was classified as having decreased sensitivity and had nucleotide base changes at four sites: 190, 198, and 199 (Fig 3). In contrast, no nucleotide differences between sensitive *M. vaginalis* were found in *MvALS1*. The gene sequence revealed that the susceptible and resistant biotypes differed by a single nucleotide.

**Fig. 3. A single base mutation in the full sequence of the *MvALS1* gene.** The red box represents nucleotide substitutions and the resulting amino acid changes.

The *MvALS1* gene sequences of seven *M. vaginalis* biotypes were compared to those of *M. vaginalis* (GenBank accession no. AB243613.1). Based on the alignment results, those nucleotide base substitutions produced 5 amino acid changes in the weed *Monochoria vaginalis* from Rawamerta and Patokbeusi that were resistant to the herbicide metsulfuron-methyl (ALS inhibitor), at positions 190 (Ser-64-Ala, TCC; sensitive, GCC; resistant), 198 (Asp-66-Glu, GAC; sensitive, GAA; resistant), 718 (Asn-240-Asp, AAT; sensitive, GAT; resistant), 1.276-1.278 (Glu-426-Asn, GAA; sensitive, AAC; resistant), and 1.406 (Ser-469-Asn. AGC; sensitive, AAC; resistant) (Fig 3). *Monochoria vaginalis* from Sukra was then subjected to three single amino acid substitutions at the same positions: 90 (Ser-64-Ala, TCC; sensitive, GCC; resistant), 198 (Asp-66-Glu, GAC; sensitive, GAA; resistant), and 718 (Asn-240-Asp, AAT; sensitive, GAT; resistant). Meanwhile, nucleotide base substitutions at positions 381 (GCG; sensitive, GCC; resistant) and 681 (CCG; sensitive, CCA; resistant) did not result in amino acid changes (Fig 3). Furthermore, the results of nucleotide base substitutions in the *MvALS1* gene showed that the *Monochoria vaginalis* biotype from Rawamerta and Patokbeusi had 5 amino acid substitutions: Ser-64-Ala, Asp-66-Glu, Asn-240-Asp, Glu-426-Asn and Ser-469-Asn and *Monochoria vaginalis* from Sukra experienced 3 single amino acid substitutions: Ser-64-Ala, Asp-66-Glu and Asn-240-Asp (Fig 4). The signal quality chromatogram detected from genotype amplicons that undergo nucleotide base substitution can be seen in S3 Fig. It implies that the above-mentioned amino acid

substitution is to blame for SU-herbicide resistance in the R biotype.

**Fig 4. Alignment of *MvALS1* gene deduced amino acid sequences.** The red box depicts amino acid changes in comparison to the susceptible biotype (Ser-64-Ala, Asp-66-Glu, Asn-240-Asp, Glu-426-Asn, and Ser-469-Asn).

The findings of this study are consistent with previous research, but at different nucleotide base positions for cases of mutations in weeds resistant to ALS enzyme inhibitor SU herbicides (bensulfuron-methyl), where the Asp-376-Glu *ALS1* amino acid substitution previously occurred in *Monochoria vaginalis* [25]. Furthermore, Ser-653-Asn has been discovered in the weeds Amaranthus hybridus (resistant to imazethapyr), Amaranthus tuberculatus (resistant to imazethapyr, pyrithiobac, and thifensulfuron), *Avena fatua* (resistant to imazamethaben and flucarbazone), and *Bromus tectorum* (resistant to imazamox) which were resistant to ALS inhibitor herbicides [26, 27, 28, 29]. This study, however, includes the first report of mutations in three other amino acids in the *MvALS1* gene: Ser-64-Ala, Asn-240-Asp, and Glu-426-Asn.

In comparison to previous research, the results of this study demonstrated the various amino acid substitution points with different positions. Previous research in Indonesia reported the amino acid substitutions Val-143-Ile and Val-148-Ile in the *ALS1* protein with a partial sequence for the first time [15]. The ALS-resistant biotype in *M. vaginalis* then experienced a single mutation in the *ALS1* gene, changing position 197 proline (CCT) to serine (TCT) in Japan [22, 25], position 376 asparagine (GAT) to glutamine (GAA) [13], and Trp-574-Leu [30].

# Discussion

The ability of metsulfuron-methyl to inhibit the formation of the ALS enzyme resulted in a decrease in weed dry weight. In *Monochoria vaginalis*, metabolism is disrupted, resulting in poisoning and even death. In some resistant weeds, inhibiting ALS enzyme formation disrupts protein formation and photosynthetic transmission, resulting in lower biomass and seed yield [31]. Weeds exposed to herbicides had a lower average reduction in weed growth based on dry weight than weeds not exposed to herbicides. A decrease in dry weight is the outcome of weed growth inhibition, which is heightened by increasing the herbicide [32].

*M. vaginalis* weeds from herbicide-resistant rice fields are more difficult to control than weeds from herbicide-free rice fields. The percentage of weed growth damage varies depending on the location and is affected by a number of variables, including the previous land use, the frequency of herbicide application by farmers, the history of land use, and the planting patterns used in each location where the weed is native [20]. The percentage of growth damage describes how much the metsulfuron-methyl herbicide dose level can reduce *M.vaginalis* growth from each area of origin. Comparing *M. vaginalis* from Rawamerta and Patokbeusi to *M. vaginalis* from other locations, the percentage of damage was lowest due to the higher intensity of herbicide application in these two areas. According to farmer interviews, rice fields in both locations use herbicides twice per planting season. Since there are three rice plants each year, herbicides are applied more frequently at this location than at others.

The level of phytotoxicity, percentage of necrotic tissue, and weed death caused by herbicides were used to assess resistance levels. As a result, minor differences such as reduced size and leaf area, to name a few, are not visible. $GR_{50}$ data is a more concrete evaluation, particularly in dose-response curves and when weeds have low levels of resistance [33]. The level of resistance must be determined because it provides an overview of the resistance mechanisms that occur, allowing appropriate strategies to be developed to control resistant weeds [14]. Weed resistance will continue to rise as monoculture practices and herbicides with the same mode of action are used [34]. Long-term herbicide use does not eliminate all weeds, and a small number of weeds will survive and evolve into resistant weed biotypes. [35]. Additionally, natural selection a process in which genetic mutations occur naturally in plants due to the continuous use of similar herbicides increasing weed resistance [36]. Differential herbicide sensitivity could be explained by differences in absorption, translocation, or metabolism rates [26].

Herbicide resistance mechanisms are classified into two types based on the level of

involvement of the target protein: target-site resistance (TSR) mechanisms and non-target-site resistance (NTSR) mechanisms. NTSR mechanisms include reduced herbicide uptake and translocation as well as increased herbicide metabolism to break down into less toxic compounds. TSR mechanisms, on the other hand, alter the amino acid sequence and expression level of the target enzyme, reducing the herbicide's ability to inhibit the enzyme or necessitating higher herbicide concentrations to achieve adequate inhibition [37]. In most cases of ALS-resistant weeds, single amino acid substitutions (TSR) have been reported. Previous research demonstrated that mutations in Pro-197 result in SU resistance [30].

Resistant weeds emerged only a few years after the introduction of sulfonylurea group herbicides (ALS inhibitors) to the herbicide market. Since then, resistant weeds have emerged, Ala122, Pro197, Trp574, or Ser653 are the most common amino acid substitutions caused by single point mutations [38, 39]. The data in (Fig 4) show that the amino acid substitution reported in the *ALS1* gene (NCBI, accession AB243613.1) is a type of resistance response to the herbicide metsulfuron-methyl (sulfonylurea) for a continuous treatment period of more than ten years in monoculture rice farming. The frequency of herbicide application and the location and type of cultivation influence the level of herbicide resistance [20].

In ALS, there is a regulatory subunit and a catalytic subunit. By producing amino acids such as valine, leucine, and isoleucine, regulatory subunits regulate catalytic subunit activity, including feedback inhibition. The herbicide cannot mimic the substrate for the enzyme, according to an analysis of the three-dimensional structures of ALS, the substrate, and the herbicide molecule. ALS inhibitor herbicides thus bind across the catalytic site of the catalytic subunit, preventing substrate access to the ALS catalytic entry point [39]. These herbicides may bind differently to the ALS protein's catalytic sites, resulting in different efficiencies for preventing these herbicide substrates from accessing the catalytic subunit. Furthermore, two chemical groups of ALS inhibitor herbicides, known as SU and TP herbicides, have been reported to bind to distinct ALS protein sites [39]. The amino acid mutations in this study, Ser-64-Ala, Asp-66-Glu, Asn-240-Asp, Glu-426-Asn, and Ser-469-Asn (Fig 4), have the potential to increase global understanding of the process and evolutionary patterns of resistance spread and help consider weed management to suppress the potential spread and evolution of resistant weeds.

This study was successful in identifying three previously unknown amino acid substitutions in the *MvALS1* gene, namely Ser-64-Ala, Asn-240-Asp, and Glu-426-Asn in the M. vaginalis biotypes Rawameta (R/S 6.00 moderate resistance), Patokbeusi (R/S 3.89 low resistance), and Sukra (R/S 2.77 decreased sensitivity). These findings suggest that resistance to metsulfuron methyl in the three *M. vaginalis* biotypes studied (Rawameta, Patokbeusi, and Sukra) was caused by the target site resistance (TSR) mechanism, specifically changes in nucleotide bases in the *MvALS1* gene. [37] discovered that non-synonymous substitutions that alter amino acid translation can result in target site resistance. Further research into resistance mechanisms from different regions leads to a more complete understanding of the evolution of the MvALS1 gene, which causes resistance in *M. vaginalis*. The condition of metsulfuron-methyl-resistant *M. vaginalis* populations from rice fields in Rawameta, Patokbeusi, and Sukra Districts, West Java, demonstrates that *M. vaginalis* is no longer effective and efficient against metsulfuron-methyl by displaying a target site resistance mechanism in the form of amino acid substitution. As a result, the integrated weed management (IWM) approach is superior for managing *M. vaginalis* resistance to metsulfuron-methyl. The IWM approach should consist of biological, mechanical, cultural, and chemical practices. Herbicide rotation and compound herbicides are two chemical options. Chinese agricultural chemical industries have developed several mixtures that are used to control *M. vaginalis* resistant populations in rice, such as penoxsulam mixed with mesotrione, cyhalofopbutyl in combination with bispyribac-sodium, and mesotrione in combination with pretilachlor. Furthermore, bentazone and fomesafen are used in combination to slow or prevent the development of ALS resistance in weeds [40]. Then, crop rotation, intercropping, allelopathy, and the use of cover crops are cultural management techniques that aid in preventing the emergence and spread of resistant weeds. Biological management can suppress weeds by increasing fertilizer dose from the recommended (150:60:40 kg $ha^{-1}$ as N:P:K) rate to 150% of the recommended rate [41], and bioorganic fertilizer was discovered to have an average weed suppression rate of more than 80% in rice

fields, making it effective in suppressing grass and broad-leaved weeds [42]. At the same time, physical control can focus on tilling the soil or Stale Seedbed practices to deplete the seed bank before planting plants [43].

## Conclusion

*Monochoria vaginalis* populations from Rawamerta were classified as moderately resistant with a resistance ratio value of 6.00, Patokbeusi as low resistance with a resistance ratio value of 3.89, and Sukra as decreased sensitivity with a resistance ratio value of 2.77 to the herbicide metsulfuron-methyl, whereas *M. vaginalis* originating from the from Patrol, East Karawang and Ciasem are classified as sensitive weeds with resistance ratios of 1.59, 1.43 and 1.36 to metsulfuron methyl, respectively. Alignment of the nucleotide bases in the *ALS1* gene reveals that the *Monochoria vaginalis* biotypes from Rawamerta and Patokbeusi experienced nucleotide substitution, resulting in 5 amino acid substitutions: Ser-64-Ala, Asp-66-Glu, Asn-240-Asp, Glu-426-Asn, and Ser-469-Asn. Then, *Monochoria vaginalis* from Sukra underwent nucleotide substitution, resulting in three amino acid substitutions: Ser-64-Ala, Asp-66-Glu, and Asn-240-Asp. In contrast, *M. vaginalis* from previous bioassays that were still classified as sensitive (Patrol, East Karawang, and Ciasem) did not show any changes in nucleotide bases. This is the first reported case of *M. vaginalis* developing resistance to metsulfuron-methyl (sulfonylurea). This also confirms for the first time that Ser-64-Ala, Asn-240-Asp, and Glu-426-Asn amino acid mutations were found in cases of *M. vaginalis* resistance to metsulfuron-methyl (ALS inhibitor).

## Supporting information

**S1 Fig. Locations of *M. vaginalis* in West Java that are suspected of being resistant to metsulfuron methyl.**
(TIF)

**S2 Fig. Visualization of the DNA quality obtained from PCR of seven different biotypes of *Monochoria vaginalis*.**
(TIF)

**S3 Fig. Chromatogram of *MvALS1* gene readings from Rawamerta, Patokbeusi, and Sukra *M. vaginalis* genotypes.**
(TIF)

**S1 Table. Purification of *M. vaginalis* results of a DNA spectrophotometer**.

(PDF)

**S2 Table. Origin Pro data result and analysis on metsulfuron-methyl $GR_{50}$.**

(PDF)


## Acknowledgments

We thank LPDP - Educational Fund Management Institution for granting the scholarship and supporting the project.


## Author Contributions

**Conceptualization:** Kansa Dianti Putri, Dwi Guntoro, Sintho Wahyuning Ardie.

**Data curation:** Kansa Dianti Putri.

**Formal analysis**: Kansa Dianti Putri.

**Funding acquisition:** Kansa Dianti Putri.

**Investigation:** Kansa Dianti Putri.

**Methodology:** Kansa Dianti Putri, Dwi Guntoro, Sintho Wahyuning Ardie.

**Project administration:** Kansa Dianti Putri.

**Resources:** Kansa Dianti Putri.

**Software:** Kansa Dianti Putri.

**Supervision:** Dwi Guntoro, Sintho Wahyuning Ardie, Hariyadi.

**Validation:** Dwi Guntoro, Sintho Wahyuning Ardie, Hariyadi.

**Visualization:** Kansa Dianti Putri.

**Writing – original draft:** Kansa Dianti Putri.

**Writing – review & editing:** Kansa Dianti Putri, Dwi Guntoro, Sintho Wahyuning Ardie.

# References


1. [BPS] Badan Pusat Statistik. Rata-rata Konsumsi Perkapita Seminggu di Daerah Perkotaan Menurut Komoditi Makanan dan Golongan Pengeluaran per Kapita Seminggu (Satuan Komoditas). 2022. Available from: https://www.bps.go.id/.

2. [BPS] Badan Pusat Statistik. Produksi Padi Menurut Kabupaten/Kota (Ton), 2021-2023. 2023. Available from: https://www.bps.go.id/.

3. Pablico PP, Moody K. A survey of lowland rice (*Oryza sativa*) weeds in Central and Southern Luzon, Philippines. Philippine Journal of Weed Science. 1985;12.

4. Guyer R, Koch W. Competitive effects of *Echinochloa crus-galli* and Monochoria vaginalis in tropical irrigated rice. InProceedings, 12th Asian-Pacific Weed Science Society Conferences. 1989 (No. 1, pp. 195-202). Asian Pacific Weed Science Society.

5. [CABI] Centre for Agriculture and Bioscience International. Invasive Species Compendium; *Monochoria vaginalis* (Pickerel Weed). 2023. Available form: https://www.cabi.org.

6. Evar, F. O, Guntoro, D, Chozin, M, Irianto, Yuli. Sulfonylurea herbicide-resistant study on broadleaf weeds in the lowland rice production center in West Java, Indonesia. Journal of Tropical Crop Science. 2021; 9(2). https://doi.org/10.29244/jtcs.9.02.137-144

7. Caton BP. A practical field guide to weeds of rice in Asia. Int. Rice Res. Inst.; 2010.

8. Beltran JC, White B, Burton M, Doole GJ, Pannell DJ. Determinants of herbicide use in rice production in the Philippines. Agricultural Economics. 2013 Jan;44(1):45-55. https://doi.org/10.1111/j.1574-0862.2012.00631.x

9. Xu Y, Xu L, Li X, Zheng M. Investigation of resistant level to tribenuron-methyl,

diversity and regional difference of the resistant mutations on acetolactate synthase (ALS) isozymes in *Descurainia sophia* L. from China. Pesticide Biochemistry and Physiology. 2020 Oct 1;169:104653. https://doi.org/10.1016/j.pestbp.2020.104653

10. Battel, Robert. How Does Herbicide Resistance Occur?. 2018. Available from: https://www.canr.msu.edu/news/how-does-herbicide- resistance-occur.

11. Jugulam M, Shyam C. Non-target-site resistance to herbicides: Recent developments. Plants. 2019 Oct 15;8(10):417. https://doi.org/10.3390/plants8100417

12. Iwakami S, Tanigaki S, Uchino A, Ozawa Y, Tominaga T, Wang GX. Characterization of the acetolactate synthase gene family in sensitive and resistant biotypes of two tetraploid Monochoria weeds, *M. vaginalis* and M. *korsakowii*. Pesticide biochemistry and physiology. 2020 May 1;165:104506. https://doi.org/10.1016/j.pestbp.2019.12.001

13. Tanigaki S, Uchino A, Okawa S, Miura C, Hamamura K, Matsuo M, Yoshino N, Ueno N, Toyama Y, Fukumi N, Kijima E. Gene expression shapes the patterns of parallel evolution of herbicide resistance in the agricultural weed *Monochoria vaginalis*. New Phytologist. 2021 Oct;232(2):928-40. https://doi.org/10.1111/nph.17624

14. Burgos NR. Whole-plant and seed bioassays for resistance confirmation. Weed Science. 2015 Feb;63(SP1):152-65. https://doi.org/10.1614/WS-D-14-00019.1

15. Widianto R, Kurniadie D, Widayat D, Umiyati U, Nasahi C, Sari S, Juraimi AS, Kato-Noguchi H. Acetolactate synthase-inhibitor resistance in *Monochoria vaginalis* (Burm. f.) C. Presl from Indonesia. Plants. 2022 Jan 31;11(3):400. https://doi.org/10.3390/plants11030400

16. Seefeldt SS, Jensen JE, Fuerst EP. Log-logistic analysis of herbicide dose-response relationships. Weed technology. 1995 Jun;9(2):218-27. https://doi.org/10.1017/S0890037X00023253

17. Originlab Crop. 2016. Origin Graphing and Analysis. [diakses 19 Oktober 2022]. https://www.originlab.com/2016.

18. Stankiewicz-Kosyl M, Haliniarz M, Wrochna M, Obrępalska-Stęplowska A, Kuc P, Łukasz J, Wińska-Krysiak M, Wrzesińska-Krupa B, Puła J, Podsiadło C, Domaradzki K. Occurrence and Mechanism of Papaver rhoeas ALS Inhibitors Resistance in Poland. Agriculture. 2022 Dec 28;13(1):82. https://doi.org/10.3390/agriculture13010082

19. Turaki AA, Ahmad B, Magaji UF, Abdulrazak UK, Yusuf BA, Hamza AB. Optimised cetyltrimethylammonium bromide (CTAB) DNA extraction method of plant leaf with high polysaccharide and polyphenolic compounds for downstream reliable molecular analyses. African Journal of Biotechnology. 2017 Jun 14;16(24):1354-65. https://doi.org/10.5897/AJB2017.15942

20. Kurniadie D, Putri KD, Widianto R, Sumekar Y, Umiyati U. Resistance test of Echinochloa crus-galli from West Java toward metsulfuron-methyl and penoxsulam. Research on Crops. 2021;22(1):53-9. https://doi.org/10.31830/2348-7542.2021.036

21. McCullough PE, McElroy JS, Yu J, Zhang H, Miller TB, Chen S, Johnston CR, Czarnota MA. ALS-resistant spotted spurge (*Chamaesyce maculata*) confirmed in Georgia. Weed Science. 2016 Jun;64(2):216-22. https://doi.org/10.1614/WS-D-15-00142.1

22. Song HY, Cho SJ, Lim HK, Park NJ, Hwang IT. Transformation a mutant *Monochoria vaginalis* acetolactate synthase (ALS) gene renders Arabidopsis

thaliana resistant to ALS inhibitors. Pesticide biochemistry and physiology. 2010 Jul 1;97(3):223-8. https://doi.org/10.1016/j.pestbp.2010.02.005

23. Spencer HJ. Metsulfuron-methyl impact on native vegetation in the Daintree lowlands, far-north Queensland, Australia. In Proceeding from the Eighteenth Australasian Weeds Conference, Weed Society of Victoria Inc, Melbourne 2012 Oct 8.

24. García MJ, Palma-Bautista C, Vazquez-Garcia JG, Rojano-Delgado AM, Osuna MD, Torra J, De Prado R. Multiple mutations in the EPSPS and ALS genes of Amaranthus hybridus underlie resistance to glyphosate and ALS inhibitors. Scientific Reports. 2020 Oct 19;10(1):17681. https://doi.org/10.1038/s41598-020-74430-0

25. Imaizumi T, Wang GX, Ohsako T, Tominaga T. Genetic diversity of sulfonylurea-resistant and-susceptible *Monochoria vaginalis* populations in Japan. Weed research. 2008 Apr;48(2):187-96. https://doi.org/10.1111/j.1365-3180.2008.00622.x

26. Whaley CM, Wilson HP, Westwood JH. ALS resistance in several smooth pigweed (*Amaranthus hybridus*) biotypes. Weed Science. 2006 Oct;54(5):828-32. https://doi.org/10.1614/WS-05-040R.1

27. Patzoldt WL, Tranel PJ. Multiple ALS mutations confer herbicide resistance in waterhemp (*Amaranthus tuberculatus*). Weed science. 2007 Oct;55(5):421-8. https://doi.org/10.1614/WS-06-213.1

28. Beckie HJ, Warwick SI, Sauder CA. Basis for herbicide resistance in Canadian populations of wild oat (*Avena fatua*). Weed Science. 2012 Mar;60(1):10-8. https://doi.org/10.1614/WS-D-11-00110.1

29. Kumar V, Jha P. First report of Ser653Asn mutation endowing high-level resistance to imazamox in downy brome (*Bromus tectorum* L.). Pest management science. 2017 Dec;73(12):2585-91. https://doi.org/10.1002/ps.4673

30. Tranel PJ, Wright TR, Heap IM. 2023. Mutations in Herbicide-Resistant Weeds to ALSInhibitors. [diakses 1 Desember 2023]. www.weedscience.com

31. Panozzo S, Scarabel L, Rosan V, Sattin M. A new Ala-122-Asn amino acid change confers decreased fitness to ALS-resistant *Echinochloa crus-galli*. Frontiers in Plant Science. 2017 Nov 28;8:2042. https://doi.org/10.3389/fpls.2017.02042

32. Majd R, Chamanabad HR, Zand E, Mohebodini M, Khiavi HK, Alebrahim MT, Tseng TM. Evaluation of herbicide treatments for control of wild gladiolus (*Gladiolus segetum*) in wheat. Applied Ecology & Environmental Research. 2019 Jun 1;17(3).http://dx.doi.org/10.15666/aeer/1703_55615570

33. Costa LO, Rizzardi MA. Resistance of *Raphanus raphanistrum* to the herbicide metsulfuron-methyl. Planta Daninha. 2014;32:181-7. https://doi.org/10.1590/S0100-83582014000100020

34. Gerhards R, Dentler J, Gutjahr C, Auburger S, Bahrs E. An approach to investigate the costs of herbicide-resistant *Alopecurus myosuroides*. Weed Research. 2016 Dec;56(6):407-14. https://doi.org/10.1111/wre.12228

35. Manik SE. Weed resistance test of *Eleusine indica* against the use of herbicide with active ingredients glyphosate (in Indonesia). AGRILAND Jurnal Ilmu Pertanian. 2019;7(1):33-8.

36. Baucom RS. Evolutionary and ecological insights from herbicide-resistant weeds: what have we learned about plant adaptation, and what is left to uncover?. New

Phytologist. 2019 Jul;223(1):68-82. https://doi.org/10.1111/nph.15723

37. Gaines TA, Duke SO, Morran S, Rigon CA, Tranel PJ, Küpper A, Dayan FE. Mechanisms of evolved herbicide resistance. Journal of Biological Chemistry. 2020 Jul 24;295(30):10307-30. https://doi.org/10.1074/jbc.REV120.013572

38. Tranel PJ, Wright TR. Resistance of weeds to ALS-inhibiting herbicides: what have we learned?. Weed science. 2002 Dec;50(6):700-12. https://doi.org/10.1614/0043-1745(2002)050[0700:RROWTA]2.0.CO;2

39. McCourt JA, Pang SS, King-Scott J, Guddat LW, Duggleby RG. Herbicide-binding sites revealed in the structure of plant acetohydroxyacid synthase. Proceedings of the National Academy of Sciences. 2006 Jan 17;103(3):569-73. https://doi.org/10.1073/pnas.050870110

40. Liu X, Merchant A, Xiang S, Zong T, Zhou X, Bai L. Managing herbicide resistance in China. Weed Science. 2021 Jan;69(1):4-17. https://doi.org/10.1017/wsc.2020.68

41. Chaudhary A, Chhokar RS, Dhanda S, Kaushik P, Kaur S, Poonia TM, Khedwal RS, Kumar S, Punia SS. Herbicide resistance to Metsulfuron-Methyl in *Rumex dentatus* L. in north-west India and its management perspectives for sustainable wheat production. Sustainability. 2021 Jun 21;13(12):6947. https://doi.org/10.3390/su13126947

42. Li Z, Li D, Zhou S, Zhou X, Bai L. The weeds control of a novel bioorganic fertilizer and its effects on agronomic traits of rice. International Journal of Agriculture and Biology. 2018;20(3):507-12.https://doi.org/10.17957/ijab/15.0509

43. Feng L, Chen GQ, Tian XS, Yang HM, Yue MF, Yang CH. The hotter the weather, the greater the infestation of *Portulaca oleracea*: opportunistic life-history traits in a serious weed. Weed Research. 2015 Aug;55(4):396-405. https://doi.org/10.1111/wre.12151

Fig1

Click here to access/download;Figure;Fig1.tiff

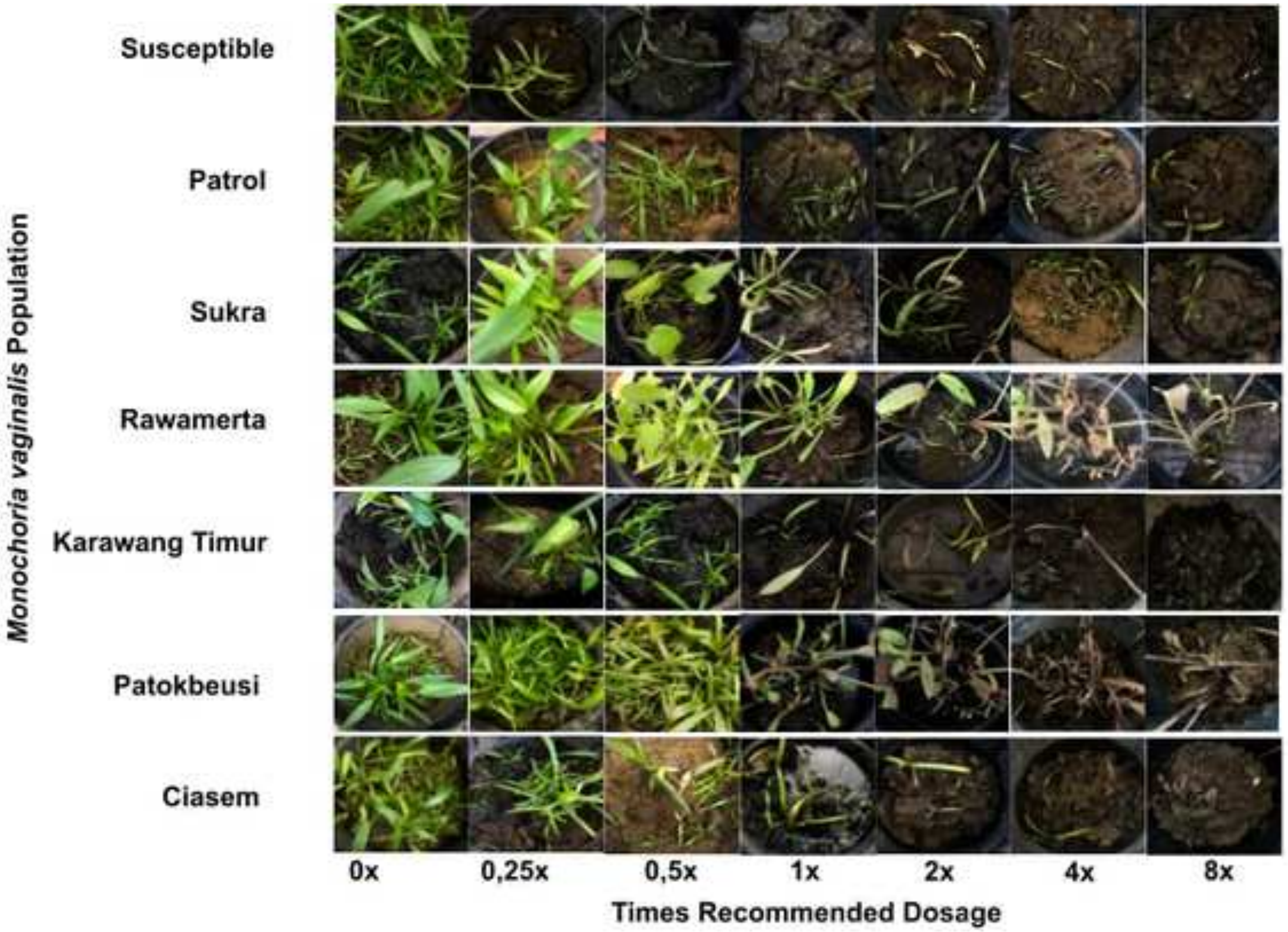

Click here to access/download;Figure;Fig2.tif

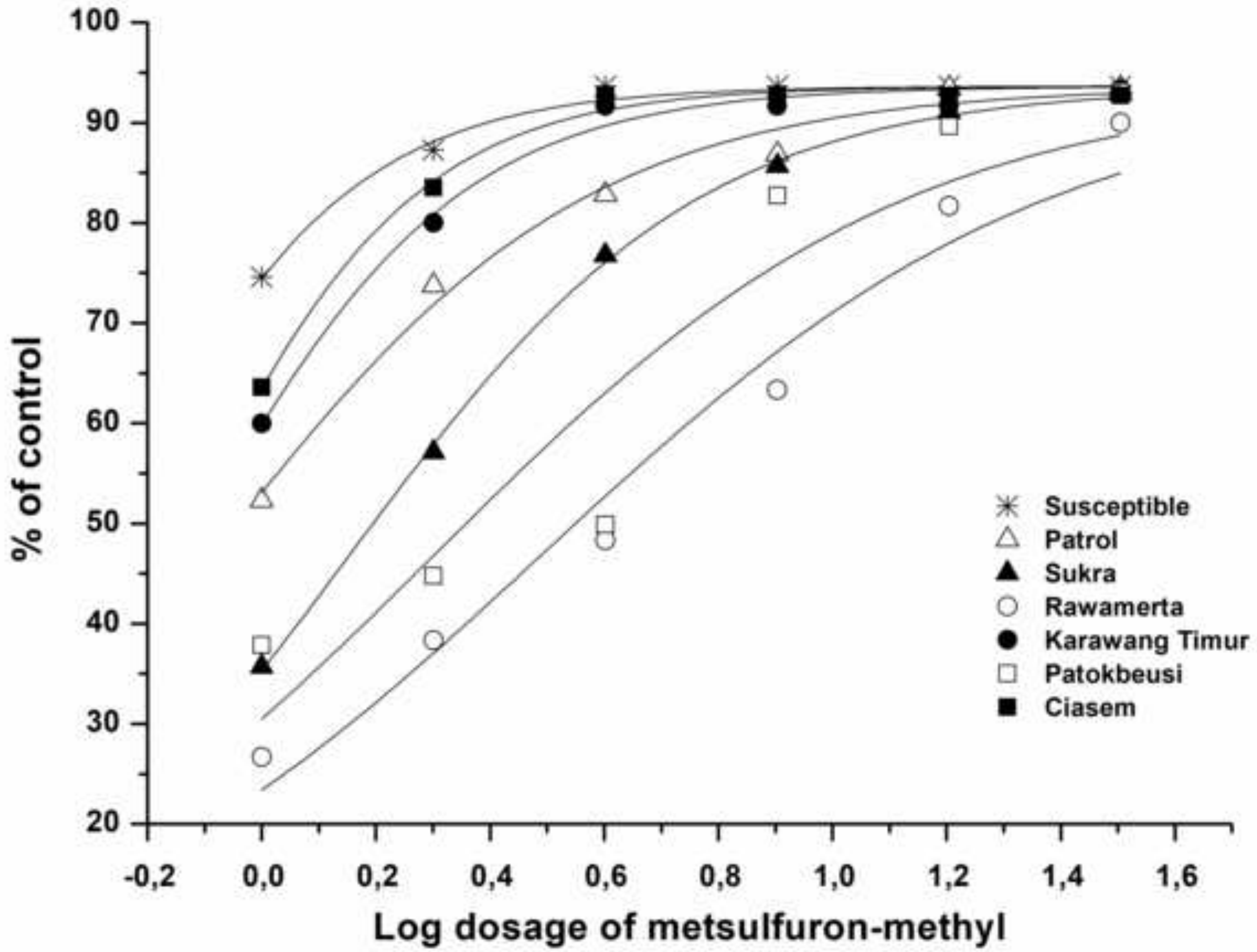

Fig3

Click here to access/download;Figure;Fig3.tiff

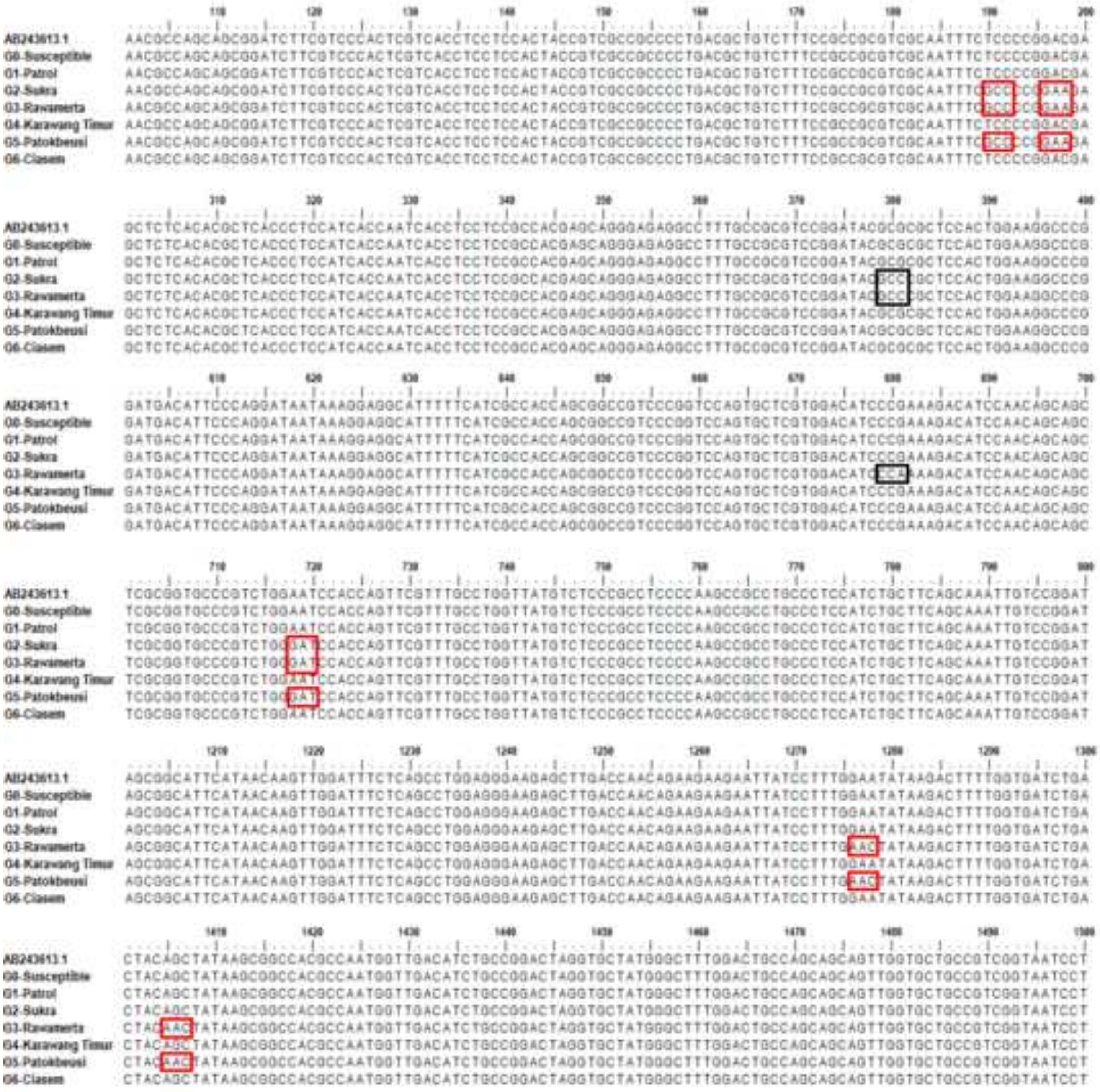

Click here to access/download;Figure;Fig4.tiff

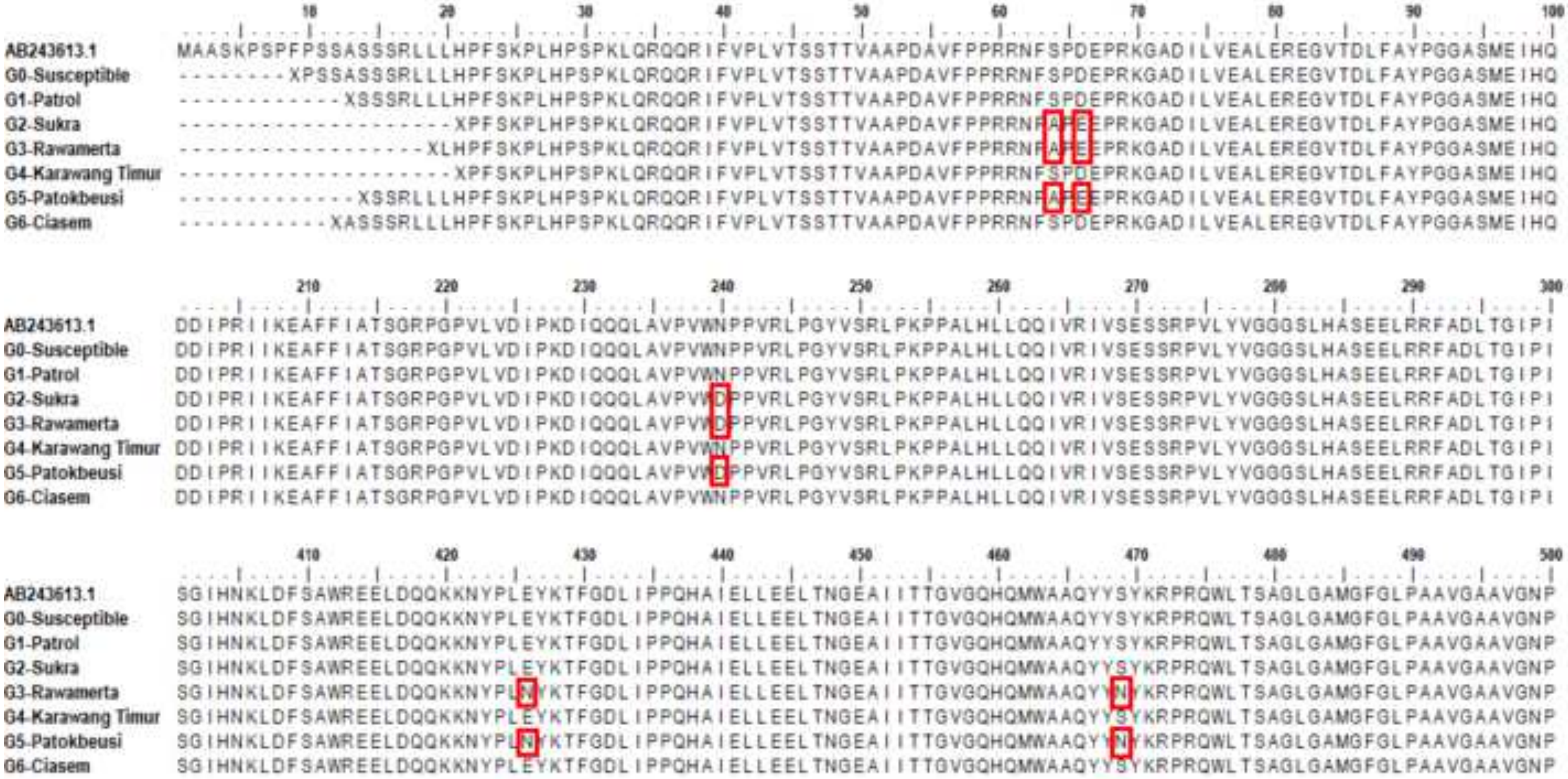

S1Fig

Click here to access/download
**Supporting Information**
S1Fig.tiff

S2Fig

Click here to access/download
**Supporting Information**
S2Fig.tiff

S3Fig

Click here to access/download
**Supporting Information**
S3Fig.tiff

S1Table

Click here to access/download
**Supporting Information**
S1Table.pdf

S2Table

Click here to access/download
**Supporting Information**
S2Table.pdf
