# Supplementary material for: A new amino acid substitution in the MvALS1 gene of metsulfuron-methyl resistant biotypes Monochoria vaginalis (Burm. f.) C. Presl from West Java, Indonesia
Source: PLoS One. 2024 Oct 4;19(10):e0308465. doi: 10.1371/journal.pone.0308465 (PMC11451974; doi:10.1371/journal.pone.0308465)
Supplement: Attachment — Submitted filename: Response to Reviewers.docx [file pone.0308465.s007.docx]

Dear Dr. Sundaram,

I hope this message finds you well. Thank you for your detailed feedback on our manuscript, PONE-D-24-02160, titled "A New Amino Acid Substitution in the *MvALS1* Gene of Metsulfuron-Methyl Resistant Biotypes *Monochoria vaginalis* (Burm. f.) C. Presl From West Java, Indonesia." We appreciate the opportunity to revise and resubmit our work. Below, we address each point raised by the academic editor and reviewers.

**Journal Requirements:**

1. Manuscript Style Requirements:

We have ensured our revised manuscript meets PLOS ONE’s style requirements, including proper file naming. We have used the provided templates:

- [PLOS ONE Formatting Sample Main Body](https://journals.plos.org/plosone/s/file?id=wjVg/PLOSOne_formatting_sample_main_body.pdf)
- [PLOS ONE Formatting Sample Title Authors Affiliations](https://journals.plos.org/plosone/s/file?id=ba62/PLOSOne_formatting_sample_title_authors_affiliations.pdf)

2. Permits Information in Methods Section:

We have included information in the Methods section regarding the fieldwork, specifying that no permits or approvals were necessary since the samples were gathered in public areas without any private or restricted access. Additionally, our sampling did not involve protected species, eliminating the need for specific conservation permits.

3. Financial Disclosure Statement:

We kindly request to update the financial disclosure statement as outlined below: This research was supported by LPDP - Indonesia Endowment Fund for Education. The funders had no role in study design, data collection and analysis, decision to publish, or preparation of the manuscript.

4. Acknowledgments Section:

We have removed any funding-related text from the Acknowledgments section.

5. Original Uncropped and Unadjusted Images:

We have ensured compliance with PLOS ONE’s requirements for blot and gel images by providing the original uncropped and unadjusted images. We have indicated in our cover letter whether these images are included in the Supporting Information.

6. Copyrighted Images in Figure 1:

We have taken the action to remove the figure 1.

7. Copyrighted Images in Figure S1:

For Figure S1, we have proceeded with the action of removing the figure from the manuscript.

8. Uploading Figure Files to PACE for Compliance Verification

We have already uploaded our figure files to the Preflight Analysis and Conversion Engine (PACE) digital diagnostic tool and are currently reviewing them. We will ensure that the figures meet PLOS requirements and will include them in the revised submission accordingly.

**Review Comments to the Author:**

Dear Dr. Simerjeet Kaur,

Thank you for your valuable feedback on our manuscript titled "A New Amino Acid Substitution in the MvALS1 Gene of Metsulfuron-Methyl Resistant Biotypes *Monochoria vaginalis* (Burm. f.) C. Presl From West Java, Indonesia," submitted to PLOS ONE under Manuscript ID PONE-D-24-02160. We appreciate the time and effort you have dedicated to reviewing our work.

We are pleased to inform you that we have carefully reviewed your comments and have addressed each point in the attached PDF document. Specifically:

1. We acknowledge your assessment regarding the significant improvements needed in the Introduction and Discussion sections. As per your comments in the attached PDF labeled PONE-D-24-02160_reviewer, we have extensively revised these sections to improve clarity and coherence.
2. Based on your suggestion, we have removed the management-related content from the Discussion section to streamline the focus on scientific findings.
3. Regarding the discussion on NTSR (Non-Target-Site Resistance) and its role in ALS resistance evolution in Monochoria, we have incorporated a detailed analysis in the Discussion section to elucidate these aspects further.
4. In the Results section, we have provided comprehensive discussions corresponding to the findings presented in Tables and Figures, ensuring clarity and relevance.

Dear Reviewer #2,

Thank you for your insightful feedback.

We have addressed your concern regarding the effects of mutations on the binding and catalytic sites by using the DUET tool to predict protein stability changes. The results of these predictions are included in the paper.

We hope this addition clarifies the impact of the mutations.

We understand the need for significant revisions based on the reviewers' comments. We have undertaken a major revision to address all points raised and ensure our manuscript meets PLOS ONE’s standards.

Thank you again for the opportunity to improve our manuscript. We look forward to resubmitting a revised version that meets PLOS ONE’s publication criteria.

Sincerely,

Kansa Dianti Putri

IPB University
